# Environmental Drivers of the Spatiotemporal Dynamics of Respiratory Syncytial Virus in the United States

**DOI:** 10.1371/journal.ppat.1004591

**Published:** 2015-01-08

**Authors:** Virginia E. Pitzer, Cécile Viboud, Wladimir J. Alonso, Tanya Wilcox, C. Jessica Metcalf, Claudia A. Steiner, Amber K. Haynes, Bryan T. Grenfell

**Affiliations:** 1 Department of Epidemiology of Microbial Diseases, Yale School of Public Health, Yale University, New Haven, Connecticut, United States of America; 2 Fogarty International Center, National Institutes of Health, Bethesda, Maryland, United States of America; 3 Department of Zoology, University of Oxford, Oxford, United Kingdom; 4 Department of Ecology and Evolutionary Biology, Princeton University, Princeton, New Jersey, United States of America; 5 Healthcare Cost and Utilization Project, Center for Delivery, Organization and Markets, Agency for Healthcare Research and Quality, US Department of Health and Human Services, Rockville, Maryland, United States of America; 6 Epidemiology Branch, Division of Viral Diseases, National Center for Immunization and Respiratory Diseases, Centers for Disease Control and Prevention, Atlanta, Georgia, United States of America; Technische Universität Dresden, Medical Faculty, Germany

## Abstract

Epidemics of respiratory syncytial virus (RSV) are known to occur in wintertime in temperate countries including the United States, but there is a limited understanding of the importance of climatic drivers in determining the seasonality of RSV. In the United States, RSV activity is highly spatially structured, with seasonal peaks beginning in Florida in November through December and ending in the upper Midwest in February-March, and prolonged disease activity in the southeastern US. Using data on both age-specific hospitalizations and laboratory reports of RSV in the US, and employing a combination of statistical and mechanistic epidemic modeling, we examined the association between environmental variables and state-specific measures of RSV seasonality. Temperature, vapor pressure, precipitation, and potential evapotranspiration (PET) were significantly associated with the timing of RSV activity across states in univariate exploratory analyses. The amplitude and timing of seasonality in the transmission rate was significantly correlated with seasonal fluctuations in PET, and negatively correlated with mean vapor pressure, minimum temperature, and precipitation. States with low mean vapor pressure and the largest seasonal variation in PET tended to experience biennial patterns of RSV activity, with alternating years of “early-big” and “late-small” epidemics. Our model for the transmission dynamics of RSV was able to replicate these biennial transitions at higher amplitudes of seasonality in the transmission rate. This successfully connects environmental drivers to the epidemic dynamics of RSV; however, it does not fully explain why RSV activity begins in Florida, one of the warmest states, when RSV is a winter-seasonal pathogen. Understanding and predicting the seasonality of RSV is essential in determining the optimal timing of immunoprophylaxis.

## Author Summary

Respiratory syncytial virus (RSV) causes annual outbreaks of respiratory disease every winter in temperate climates, which can be severe particularly among infants. In the United States, RSV activity begins each autumn in Florida and appears to spread from the southeast to the northwest. Using data on hospitalizations and laboratory tests for RSV, we show that the timing of epidemics is associated with a variety of climatic factors, including temperature, vapor pressure, precipitation, and potential evapotranspiration (PET). Furthermore, using a dynamic model, we show that seasonal variation in the transmission rate of RSV can be correlated with the amplitude and timing of variation in PET, which is a measure of demand for water from the atmosphere. States with colder, drier weather and a large seasonal swing in PET tended to experience an alternating pattern of “early-big” RSV epidemics one year followed by a “late-small” epidemic the next year, which our model was able to reproduce based on the interaction between susceptible and infectious individuals. However, we cannot fully explain why epidemics begin in Florida. Being able to understand and predict the timing of RSV activity is important for optimizing the delivery of immunoprophylaxis to high-risk individuals.

## Introduction

Respiratory syncytial virus (RSV) is a major cause of severe lower respiratory tract infections, including bronchiolitis and pneumonia. Most children experience their first infection by age 2 years, but immunity is imperfect and infections continue to occur throughout life, although subsequent infections tend to be less severe. An estimated 66,000–199,000 deaths in children <5 years old are associated with RSV globally, the majority occurring in developing countries [1]. In the United States (US), RSV remains a major cause of severe respiratory infection in infants <1 year of age, and has been estimated to cause >2,000 hospitalizations per 100,000 infants per year [2]. The incidence of RSV is strongly seasonal in the US and other temperate countries, with the majority of cases occurring during annual winter epidemics [3]–[8]. However, the timing of RSV activity varies substantially among different regions of the US, with year-round circulation and peak activity as early as October in southeastern Florida [9], and peaks occurring as late as May in the upper Midwest [4], [5], [10].

The development of a safe and effective vaccine against RSV has proven difficult. Therefore, prevention relies upon passive immunoprophylaxis with palivizumab to reduce the number of severe outcomes associated with RSV infection among high-risk infants [11]. While palivizumab is effective at lessening the severity of RSV infections in certain infants and children, the treatment is very expensive and the protection afforded is short-lived, requiring monthly injections during the RSV season; therefore, predicting the timing of RSV activity is essential to optimizing the cost-effectiveness of immunoprophylaxis [12]–[14].

Attempts have been made to correlate RSV seasonality with climatic variables [13], [15]–[25]. Such phenomenological analyses are important; however, RSV epidemics show strong signatures of nonlinear epidemic dynamics [26]–[29] and few studies have explored climatic associations in a variety of locations covering a range of RSV seasonality patterns and climatic regimes [25]. Therefore, we begin with linear models relating incidence patterns and candidate environmental drivers of RSV epidemics in US states. We then refine these to account for the dynamics of infection and fluctuations in immunity and susceptibility that can influence the relationship between environmental factors and epidemic timing [29]–[33].

By combining statistical analyses with mathematical modeling of the transmission dynamics of RSV across the US, we aimed to gain a better understanding of the important drivers of the spatiotemporal pattern of RSV epidemics. We build upon previous efforts to model the transmission dynamics of RSV [26]–[30], [34], [35] by fitting our model to data from a large number of states with similar underlying socio-demographics, but markedly different climatic conditions. By analyzing the relationship between estimated seasonality parameters and climatic variables, we are able to shed light on the environmental drivers of RSV transmission.

## Results

### Description of spatiotemporal patterns of RSV

**Figure 1:**
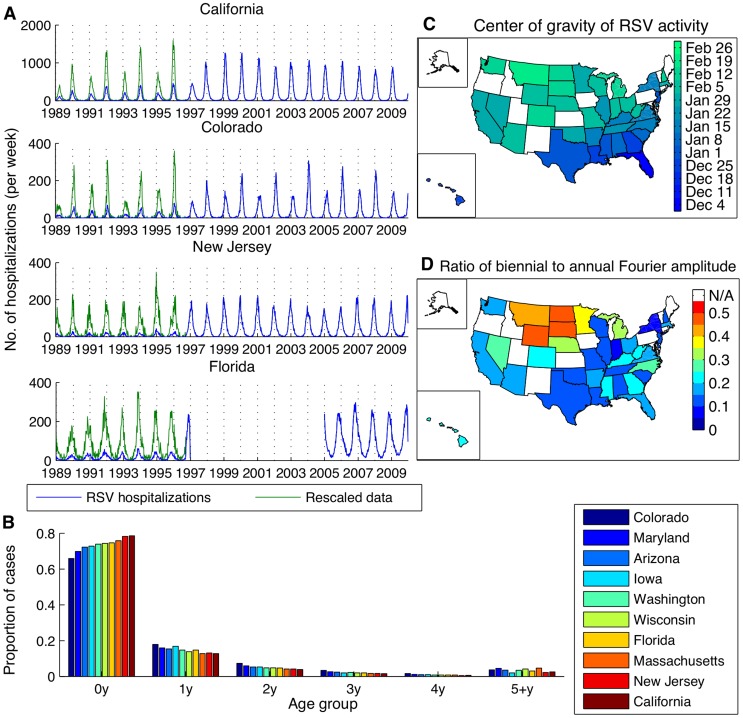
Patterns of RSV activity across the United States for hospitalization and laboratory testing data. (*A*) Time series of weekly RSV hospitalizations in select states. Raw hospitalization data is shown in blue, while the rescaled data accounting for the addition of an RSV-specific ICD-9 code in September 1996 is shown in green. (*B*) Age distribution of RSV hospitalizations across ten states. (*C*) Center of gravity of RSV activity in states with at least ten consecutive years of laboratory reports. (*D*) Strength of biennial cycle in RSV activity, as indicated by the ratio of the biennial to annual Fourier amplitude for laboratory report data.

Hospitalizations for RSV were strongly seasonal, with annual epidemics occurring during the winter months in most states (Fig. 1A, S1 Fig.). Some states (e.g. Colorado, Iowa, California in the 1990s) exhibited biennial patterns of alternating “early-big” epidemics in/around January of even-numbered years and “late-small” epidemics in/around February of odd-numbered years. The peak in RSV hospitalizations was notably earlier in Florida (occurring in November/December) compared to the other states, and hospitalizations occurred throughout the year (Fig. 1A). The vast majority (>97%) of RSV-coded hospitalizations occurred among children <5 years of age, and ∼75% occurred among children <1 year of age. The age distribution of cases varied slightly by state (Fig. 1B).

Laboratory reports of RSV-positive specimens exhibited a distinct spatial pattern, with mean timing of RSV activity (as indicated by center of gravity, a measure of mean epidemic week (S1 Text) [36]) occurring earliest in Florida and latest in Montana (Fig. 1C). Again, some states exhibited a biennial pattern of RSV epidemics; these states were highly concentrated in the upper Midwest and West regions (Fig. 1D). The laboratory and hospitalization data were highly correlated for those states with both types of data available (r>0.71, *p*<0.0001, S1 Table).

### Linking environmental drivers and timing of RSV activity

We explored trends between a variety of climatic and non-climatic variables and timing of RSV activity across US states, as measured by both center of gravity and phase difference with Florida (Table 1 and S2 Table, S2 Fig.). Negative associations were found with annual mean vapor pressure, temperature, precipitation, and potential evapotranspiration (PET), and were generally stronger when considering the mean value for the fall months (September-November) for each climatic factor. Population size and latitude were also associated with RSV timing (Table 1). Fall vapor pressure had the highest explanatory power (*R* ^2^ = 72–76%), and was also the only significant factor in an exploratory multivariate analysis (*p*<0.0001) (S3 Table). Note that while these analyses may be indicative of statistical trends, they do not account for the intrinsic nonlinear epidemic dynamics of RSV.

**Table 1:** Univariate regression of timing of RSV activity in 50 US states and District of Columbia, 1989–2010, against monthly climatic, population and geographic indicators.

	Model explaining phase timing				Model explaining gravity timing			
Explanatory variable	Parameter estimate (SE)	R^2^	Parameter estimate (SE)	R^2^	Parameter estimate (SE)	R^2^	Parameter estimate (SE)	R^2^
*Climate variables:*	Annual average		Fall average^a^		Annual average		Fall average	
**Vapor pressure**	−0.065*** (0.006)	**67%**	−0.064*** (0.006)	**72%**	−0.58*** (0.05)	**72%**	−0.57*** (0.04)	**76%**
**Temperature** b	−0.049*** (0.005)	**46**–**63%**	−0.05*** (0.005)	**45**–**64%**	−0.45*** (0.04)	**53**–**72%**	−0.46*** (0.04)	**55**–**73%**
**Precipitation**	−0.006*** (0.001)	**35%**	−0.005*** (0.001)	**34%**	−0.05*** (0.009)	**43%**	−0. 05*** (0.009)	**39%**
**Potential evapotranspiration**	**−0.12** * **(0.06)**	**6%**	**−0.14** * **(0.06)**	**8%**	**−1.15** * **(0.05)**	**8%**	−1.39** (0.52)	**11%**
Wet days	−0.013 (0.015)	0%	−0.004 (0.015)	0%	−0. 13 (0.13)	0%	−0.03 (0. 13)	0%
Cloud cover	0.006 (0.006)	0%	0.006 (0.005)	0%	0.05 (0.05)	0%	0.07 (0.04)	3%
Diurnal temperature range	0.04 (0.02)	5%	0.04 (0.02)	5%	0.34 (0.19)	4%	0.29 (0.18)	3%
*Non-climate variables:*								
**Pop size**	**−1.42 E-8** * **(6.4 E-9)**	**7%**			−1 E-7 (6 E-8)	4%		
Pop density	−0.00004 (0.00003)	2%			−0.00036 (0.00024)	2%		
**Latitude**	0.03*** (0.005)	**44%**			0.30*** (0.04)	**53%**		
Longitude	−0.003 (0.002)	1%			−0.030 (0.018)	1%		
Sampling (# RSV tests)	−6 E-7 (4E-7)	0%			−5 E-6 (4E-6)	1%		

Separate models are built for phase timing (average weekly phase difference with Florida, the earliest RSV state) and center of gravity (weighted average of RSV epidemic week, where each week is weighted by the number of RSV cases). All epidemic measures are based on weekly laboratory-reported RSV time series. Boldface indicates significance (*p*<0.05) in the exploratory analysis.

* *p*<0.05; ** *p*<0.01; *** *p*<0.0001

a Average value for September, October, and November.

b Minimum, maximum and average monthly temperatures were considered. R^2^ represents the range for the 3 variables. In all models, minimum temperature was the most strongly associated with RSV timing; hence parameter estimates listed in this table are for minimum temperature.

### Dynamic modeling analyses

**Figure 2:**
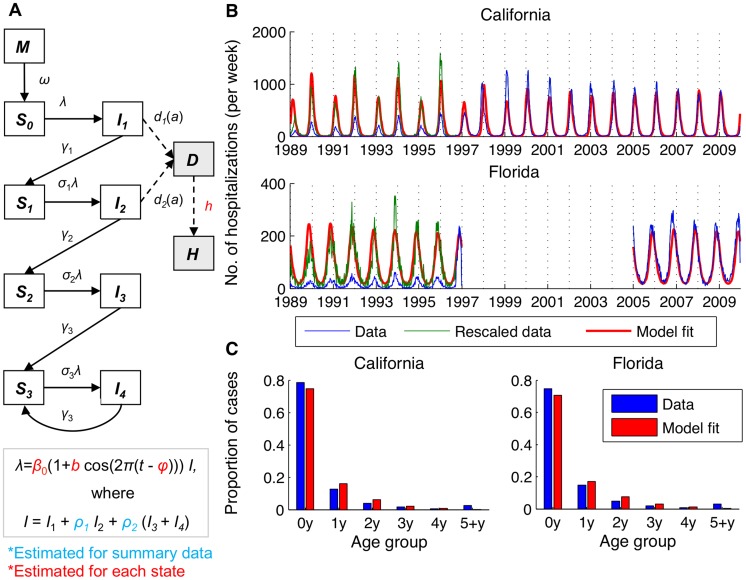
Transmission dynamic model for RSV and fit to age-specific hospitalization data. (*A*) Compartmental diagram illustrating the structure of the model. White boxes represent infection states in the model, while grey boxes represent diseased/observed states (severe lower respiratory disease, *D*, and observed cases, *H*). (*B*) Model fit to weekly RSV hospitalization data for California and Florida. The ICD9-CM coded hospitalization data is shown in blue, the rescaled data is shown in green, and the fitted models are shown in red. (*C*) Age distribution of RSV hospitalizations in California and Florida for hospitalization data and fitted models.

Mathematical modeling of the transmission dynamics of RSV allows us to explore the mechanistic relationship between the potentially important environmental variables and seasonal variation in the *transmission rate*, via which the environmental variables would likely act to affect the incidence of RSV [30]. We developed an age-stratified SIRS (Susceptible-Infectious-Recovered-Susceptible) model for the transmission dynamics of RSV, accounting for repeat infections and using natural history parameters derived from RSV cohort studies (Table 2). The model was able to reproduce the age distribution (*χ* ^2^<0.17, *p*<0.005) and seasonal pattern of RSV hospitalizations in ten states (correlation between observed and predicted annual center of gravity: *r* = 0.87, *p*<0.005) (Fig. 2, S1 Fig.). Notably, the model was able to reproduce the biennial pattern of epidemics evident in some states even though we assume that the transmission rate of RSV follows the *same* seasonal pattern every year. Furthermore, the model was able to replicate the transition from biennial epidemics during the 1990s to annual epidemics during the 2000s that occurred in California, possibly due to changes in the birth rate.

**Table 2:** Transmission dynamic model parameters.

Parameter description	Symbol	Parameter value	Source
Duration of maternal immunity	1/*ω*	16 weeks	[65]
Duration of infectiousness			
* First infection*	1/*γ*_1_	10 days	[71], [72]
* Second infection*	1/*γ*_2_	7 days	
* Subsequent infection*	1/*γ*_3_	5 days	
Relative risk of infection following			
* First infection*	*σ*_1_	0.76	[37], [66]–[68]
* Second infection*	*σ*_2_	0.6	
* Third infection*	*σ*_3_	0.4	
Proportion of infections leading to lower respiratory tract infection			
* First infection, <6 months old*	*d_p_*_,0_	0.5	[37], [66], [69]
* 6*–*11 months old*	*d_p_*_,0.5_	0.3	
* 1*–*2 years old*	*d_p_*_,1_	0.2	
* ≥2 years old*	*d_p_*_,2_	0.1	
* Second infection*	*d_s_*_,*a*_	= 0.75*d_p,a_	
Relative infectiousness			
* Second infections*	*ρ*_1_	0.75	[37], [66], [69]
* Subsequent infections*	*ρ*_2_	0.51	Estimated

From the best-fitting model to the aggregate data from the nine states with complete age-stratified hospitalization time series from 1989–2009, we estimated the relative infectiousness of third and subsequent infections compared to first two infections to be 0.51 (Table 2). The mean value of *R*_0_ was estimated to be 8.9, but we observed state-specific variation in *R*_0_ (with estimated values between 8.9 and 9.2), which was significantly correlated with population density (*r* = 0.77, *p*<0.01) (S3 Fig.). The estimated hospitalized fraction (*h*) also varied among states (from 3.2% in California to 6.9% in Colorado), but was not significantly correlated with population size or density, nor were estimates of *R*_0_ and *h* significantly correlated with one another (S4 Table). Our estimates of the hospitalized fraction are similar albeit slightly lower than the 7–8% of infants with lower respiratory tract infections who were hospitalized during cohort studies conducted in the US and Kenya [37], [38]; this is not surprising given one US-based study noted that only 45% of RSV-positive inpatients received an RSV-associated diagnosis [39].

The amplitude and timing of sinusoidal seasonal variation in the transmission rate estimated by fitting the model to the hospitalization data were both negatively correlated with mean vapor pressure and mean precipitation (*p*<0.01), and positively correlated with the amplitude and timing of PET in each state (*p*<0.01) (Table 3). The seasonal offset parameter (illustrating timing of peak transmissibility) was also significantly correlated with mean minimum temperature (*p*<0.01). These parameter estimates were also positively correlated (*p*<0.001) (S4 Table).

**Table 3:** Correlation coefficients between climatic variables and estimated seasonality parameters in RSV transmission model.

	Hospitalization data		Laboratory data	
Climatic variable	*Amplitude of seasonality (b)*	*Seasonal offset (φ)*	*Amplitude of seasonality (b)*	*Seasonal offset (φ)*
Vapor pressure				
* Mean*	−0.832*	−0.942***	−0.788***	−0.862***
* Amplitude*	−0.511	−0.600	−0.307	−0.437*
* Offset*	0.404	0.085	−0.341	−0.112
Minimum temperature				
* Mean*	−0.575	−0.801*	−0.760***	−0.782***
* Amplitude*	0.253	0.201	0.469*	0.404
* Offset*	−0.114	−0.431	−0.341	−0.119
Precipitation				
* Mean*	−0.844*	−0.774*	−0.760***	−0.733***
* Amplitude*	−0.305	−0.201	−0.036	0.066
* Offset*	−0.090	−0.313	0.213	0.092
Potential evapotranspiration				
* Mean*	0.212	−0.030	−0.104	−0.184
* Amplitude*	0.810*	0.699	0.689***	0.671***
* Offset*	0.802*	0.930**	0.611***	0.787***
Wet days				
*Mean*	−0.537	−0.361	−0.487*	−0.256
* Amplitude*	−0.527	−0.236	−0.053	0.074
* Offset*	0.033	−0.112	−0.088	−0.279
Cloud cover				
* Mean*	−0.388	−0.134	−0.091	−0.006
* Amplitude*	0.385	0.479	0.470*	0.588**
* Offset*	−0.824*	−0.812*	−0.554**	−0.755***
Diurnal temperature range				
* Mean*	−0.388	−0.134	0.522**	0.355
* Amplitude*	0.084	0.284	0.574**	0.493*
* Offset*	0.745	0.874**	0.582**	0.736***

*p<0.01, **p<0.001, ***p<0.0001.

Fitting our model to the laboratory surveillance data for RSV allowed for a more extensive analysis of the relationship between state-specific climate indicators and the amplitude and timing of seasonal variability in the transmission rate across a large number of states with different climates. Since the laboratory data did not contain the age of cases, we estimated *R*_0_ for each state based on the observed relationship between *R*_0_ and population density prior to fitting the model.

**Figure 3:**
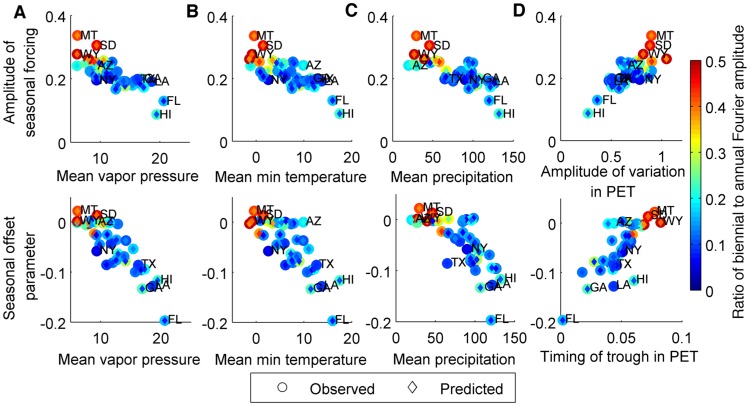
Relationship between estimated seasonality parameters for model fit to laboratory report data and select climatic factors. The estimated amplitude of seasonal forcing in RSV transmission (top) and the estimated seasonal offset parameter (bottom: *φ* = 0 represents January 1 and *φ* = −0.2 represents October 19) is plotted against (*A*) annual mean vapor pressure (hecta-Pascals), (*B*) annual mean minimum temperature (°C), (*C*) annual mean precipitation (mm/month), and (*D*) amplitude (relative to the annual mean) and timing of trough in potential evapotranspiration (PET; 0 = January 1, 0.1 = February 6). The colorbar on the right indicates the ratio of the biennial to annual Fourier amplitude for the observed data (outer circle) and fitted model (inner diamond). Select states are labeled: Arizona (AZ), Florida (FL), Georgia (GA), Hawaii (HI), Louisiana (LA), Montana (MT), New York (NY), South Dakota (SD), Texas (TX), Wyoming (WY).

Again, we found significant negative correlations between the amplitude and peak timing of RSV seasonal forcing (i.e. seasonality in the transmission rate) and the mean vapor pressure, minimum temperature, and precipitation across the 38 states (*p*<0.0001) (Table 3, Fig. 3A-C), i.e. warmer, wetter states tended to exhibit less seasonal variation and an earlier peak in the transmission rate of RSV than cooler, drier states. We also found a weaker but still significant positive correlation between the amplitude of seasonal forcing and the amplitude of variation in minimum temperature (*p*<0.005). Estimates for peak RSV transmissibility, however, were not correlated with the timing of the seasonal trough in minimum temperature or vapor pressure (Table 3). A strong and significant positive correlation was observed between both the amplitude and peak timing of RSV seasonal forcing and the seasonal variation in PET (*p*<0.0001) (Table 3, Fig. 3D). However, the state-to-state variability in the timing of peak RSV transmissibility was more than twice the observed variability in the timing of PET troughs (Fig. 3D).

The model was again able to capture the biennial pattern of RSV epidemics apparent in some states. The correlation between the observed and predicted ratio of the biennial to annual periodicities, as estimated by Fourier analysis, was 0.89 (*p*<0.0005). States with biennial RSV dynamics tended to have strong seasonal forcing (*b*>0.25), which was associated with a large amplitude of variation in PET and low minimum temperature, vapor pressure, and precipitation (Fig. 3). In general, the ratio of the biennial to annual Fourier amplitude was slightly greater in the data than predicted by the models; this is likely due to random year-to-year variability in the size of RSV epidemics, which is not accounted for in our deterministic models.

It was not possible to explain unusually high or low RSV activity within a given state, apart from the regular biennial patterns, based on any of the climatic variables. Deviations from model-predicted patterns (observed minus predicted monthly RSV lab reports) were not significantly correlated with temperature, vapor pressure, precipitation, or PET (*p*>0.05) (S4 Fig.). Furthermore, we were not able to explain year-to-year variation in epidemic timing and size by directly parameterizing variation in the transmission rate based on weekly variations in PET (S1 Text). Such a model also provided a poor fit to the data, as indicated by the log-likelihood (S5 Table).

## Discussion

The spatiotemporal pattern of RSV activity in the United States is in stark contrast to that of influenza [40] and rotavirus [36], despite the fact that all are imperfectly immunizing infections that exhibit strongly winter-seasonal epidemics. Using a combination of exploratory statistical analyses and dynamic modeling approaches, we set out to better understand geographical differences in RSV seasonality and periodicities across the US and pinpoint meaningful associations with climate. Identifying causal relationships between climatic variables and RSV patterns could be used to build predictive models of RSV incidence, which would help inform guidelines for timing of immunoprophylaxis. Our results indicate that climatological factors, particularly in vapor pressure, minimum temperature, precipitation, and PET, are strong candidates to explain the seasonal pattern of RSV epidemics in the United States. States with low mean vapor pressure, minimum temperature, and precipitation and large seasonal variation in PET tended to exhibit a later peak in the timing of RSV transmission and stronger seasonal forcing, potentially leading to biennial epidemics.

**Figure 4:**
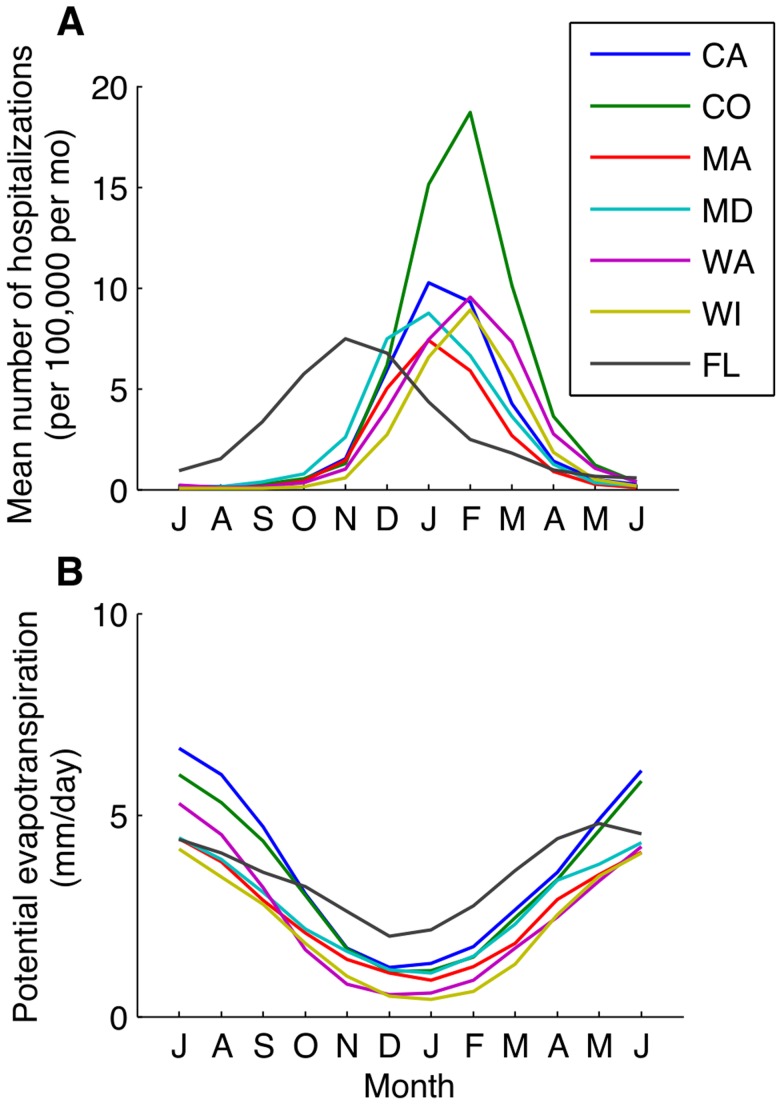
Monthly patterns of RSV activity and potential evapotranspiration. (*A*) The mean number of RSV hospitalizations per 100,000 total population per month for select states, beginning in July. (*B*) The monthly mean potential evapotranspiration (mm/day) is plotted for each state.

Seasonal variation in PET was more tightly linked to seasonal variability in the transmission rate of RSV compared to other climate factors. Potential evapotranspiration is a measure of the demand for water from the atmosphere, and tends to be highest during the summer and lowest during the winter months (Fig. 4B and S5 Fig.). RSV is transmitted via droplets and respiratory secretions, and tends to be rapidly inactivated in small aerosols [41]. Therefore, PET may be correlated with the drying time of respiratory secretions and thus virus survival, but more research is needed in this regard.

### Impact of climatic drivers on RSV

A number of studies have examined the statistical association between climatic factors and RSV seasonality. Most studies have found a significant association between temperature and RSV activity using time series correlation; however, the direction of the association is not consistent across studies, and such studies do not control for the temporal dependence among observations. RSV activity tends to occur in the coldest months in temperate regions where winter outbreaks are common [6], [13], [18]–[20], [22], [42] and in the warmest months in subtropical and tropical climates [16], [25]. Low absolute humidity (proportional to vapor pressure) has been found to be an important correlate of RSV activity in Spain [20]. Yusuf et al. [25] also observed significant correlations between dew point (a measure of absolute humidity) and RSV activity across nine cities worldwide, but again the association was negative in temperate locations and positive in subtropical locales. Paynter et al. [30] used a mathematical model to show that seasonality in the transmission rate of RSV followed a similar pattern to rainfall in the Philippines. Accordingly, the peak in RSV activity coincides with the rainy season in a number of tropical locations [15], [43]. It may be that both colder temperatures and rain cause people to aggregate indoors, thereby facilitating RSV transmission [6], [44]. More studies are needed to tease apart the associations between climate, human behavior, and infectious disease transmission. Interestingly, our study is the first to explore the association between PET and RSV dynamics.

Our US analysis indicates that mean vapor pressure, minimum temperature, precipitation, and seasonal variation in PET are good candidates to explain the timing of RSV activity across different states in the US. However, states with higher minimum fall temperature and vapor pressure tend to experience earlier RSV activity, which is difficult to reconcile with the broad seasonal patterns of this pathogen, which predominates in winter in temperate regions. Furthermore, the estimated seasonal offset parameters (i.e. timing of peak transmission) did not correspond well with the timing of the seasonal trough in temperature and vapor pressure across states. In contrast, the association between RSV and PET is more consistent, as states with lower seasonal variation in PET, lower average PET, and earlier wintertime PET troughs tend to experience earlier and less strongly seasonal RSV epidemics (Florida being the most extreme example). However, as most climatic variables are typically highly correlated, it is difficult to pinpoint a single driver of RSV dynamics with great certainty. Climate variables may be a proxy for something else that affects transmission, or there may be a more complex relationship between climate and RSV transmission (e.g. non-linear or threshold effects). Furthermore, most climatic conditions (including PET) will vary between indoor and outdoor environments. Additional analyses using similar methodological approaches in different geographic settings and at different geographic scales may be able to further disentangle the effect of various climatic factors.

It is possible that the influence of climatic factors on RSV seasonality may be modulated by other important factors that affect the RSV transmission. Demographic factors such as population density and crowding indices have been shown to be associated with the length of the RSV season across different parts of Colorado [45]. Birth rates have been shown to be an important driver of the spatiotemporal pattern of rotavirus epidemics in the United States [36], but do not appear to be correlated with the timing of RSV epidemics. Changes in the birth rate in California (which tended to be larger than within other states) may help to explain why epidemics in this state transitioned from biennial during the 1990s to annual during the 2000s; however, more work is needed to understand how variation in birth rates and transmission rates may impact RSV dynamics. Increased contact rates among older siblings during school terms have also been hypothesized to play an important role in determining RSV seasonality [13], [46], and have been shown to play in important role for other respiratory infections [47]–[49]. However, we found that incorporating school-term forcing did not improve the fit of our model to the data (S1 Text, S6 Table). By modeling the transmission dynamics of RSV, we are able to account for how these factors may interact with other sources of seasonal forcing, such as climatic factors, while controlling for the temporal dependence in the data.

### Relationship to previous mathematical models for RSV

Our findings are similar to those of other dynamic models of RSV. White et al. [27] and Weber et al. [29] found that the estimated amplitude of seasonal forcing was generally greater for temperate locations and could explain the biennial pattern of RSV epidemics in Turku, Finland. These biennial epidemic patterns, in particular, cannot be explained by statistical associations alone; they result from the dynamic feedback that occurs when an annual seasonal forcing leads to an “overshooting” of the susceptible population in the big epidemic years, leaving fewer susceptible individuals who can be infected the following year. We build on these previous analyses by fitting our model to a large number of states with different climates. As such, we are able to correlate differences in the estimated seasonal forcing parameters for various states to climatological differences. Our estimates of the amplitude of seasonal forcing are greater than those of the best-fit model of White et al. [27], but similar to those of Weber et al. for Florida and sites that exhibit biennial epidemics (e.g. Montana and Finland) [29].

Fitting our model to the age-specific hospitalization data gave us more power to estimate *R*_0_ compared to previous dynamic models, which were fit to data aggregated across age groups. Our estimates of *R*_0_ = 8.9–9.2 were similar to those obtained by White et al. (2007) for their best-fitting model, and were slightly higher than those obtained by Weber et al. (2001) for a similar model structure. Any discrepancies can likely be explained by differences in parameter assumptions. For example, unlike Weber et al. (2001), we assume a decrease in the duration of infectiousness and severity of repeated infections. This relatively high value of *R*_0_ suggests that controlling RSV transmission will require substantial effort.

Climate change, in particular an increase in average annual temperature, has been hypothesized to be responsible for shortening the RSV season in England [17]. However, a shift towards earlier RSV epidemics has also been observed in São Paulo, Brazil, which cannot be attributed to changes in climate [13]. A similar shift towards later epidemics has been noted elsewhere [45]. We also observed some changes in the timing of RSV activity in the US, mostly towards earlier RSV activity, but these patterns do not appear to be linked to climate trends (S7 Table).

### Caveats and future directions

An important limitation of our analyses is that we did not have data on the genetic strains of RSV causing cases over time and among different states. Therefore, we did not explicitly model the interaction of RSV types A and B, which White et al. (2005) found could help explain differences in the transmission dynamics of RSV in England and Wales and Turku, Finland. It is possible that strain cycling may help to explain between-year deviations from model-predicted epidemic patterns, for which we did not find any correlation with climatic factors (S4 Fig.). However, the interaction among different subtypes of RSV is unlikely to be the main driver of differences in the seasonality and periodicity of RSV activity among different states. In cities with biennial RSV activity, a single subtype has been observed to predominate for two years (i.e. through both an early-big and late-small season) prior to being replaced by the other subtype [50]–[52]. Furthermore, no association was observed between subtype predominance and epidemic severity and timing over 15 years in one US city [53].

Another limitation is that we assumed age-specific population mixing patterns were equivalent to those in the Netherlands [54], since no such broadly based studies have been conducted in the United States. Population mixing patterns were broadly similar across a variety of European countries [55], and are likely to be similar in the United States. However, the Netherlands appears to have slightly higher contact rates among 0–4 year olds compared to other countries such as the United Kingdom [54], [56]. This may have influenced our estimates of *R*_0_, as well as limited the potential impact of school-term forcing in helping to explain the spatiotemporal pattern (S1 Text). Similar studies of population mixing, and how such patterns vary seasonally, should be conducted in the United States. Also, the lack of age detail in the laboratory report data limited our ability to directly estimate *R*_0_ for all states included in the analysis.

Finally, our epidemiological datasets were prone to changes in reporting and coding practices, and these datasets captured only a fraction of all RSV infections. We elected to use very specific RSV outcomes to have an accurate picture of the age distribution of RSV cases, and addressed sensitivity issues by rescaling the data to remove time trends and incorporating an estimated reporting rate in our transmission model. Importantly, sensitivity analyses indicate that our results are robust to the rescaling method and that RSV-specific hospital admissions align well with broader outcomes such as bronchiolitis (S8 Table, S6–S7 Fig.).

One potential hypothesis that follows from this study, which may help to explain why RSV activity in the US begins in Florida (particularly southeastern Florida [9]), is that less variable climatic conditions in Florida combined with high population density in cities such as Miami allows for year-round persistence of RSV. In contrast, other states may experience routine fadeouts of RSV infection during the summer months. If this were the case, then peak RSV activity in Florida could begin as soon as climatic conditions (and population mixing) favored a slight increase in transmissibility of the virus, whereas other states may be dependent on outside introduction of the virus once the effective reproductive number (*R_e_* = *R*_0_ x the proportion susceptible) is greater than 1. Indeed, White et al. (2007) observed frequent fadeouts of infection in a stochastic version of their best-fitting model, particularly in temperate settings. The role of fadeouts in the spatiotemporal dynamics of RSV could be explored using a stochastic metapopulation model informed by local rather than state-aggregate data, which is an important direction for future research. The availability of detailed data and large variability in climate make the United States a very useful test case for this. Further, it would be interesting to compare the results with Europe, where strain typing has also been performed. Finally, a more detailed understanding of the spatial transmission of this disease could be obtained by fitting phylogeographic models to large-scale viral sequencing data [57]. Such models were instrumental in elucidating the migration patterns of the influenza virus over the past decade [58], but require a large amount of well-sampled molecular data, which are not yet available for RSV.

We have been able to demonstrate that mean vapor pressure, temperature, and precipitation as well as seasonal fluctuations in PET are correlated with seasonal variation in the transmission rate of RSV; these factors could help to explain differences in the strength of RSV seasonality across the different regions of the United States. Stronger seasonal forcing can also drive the occurrence of biennial patterns of RSV activity. However, the rationale behind why RSV epidemics tend to begin in the southeastern United States remains elusive. Our analysis highlights the role of potential evapotranspiration as a previously unidentified correlate of RSV transmission. A better understanding of the relationship between PET and RSV survival may help predict the timing of RSV activity across the United States and further guide the optimal timing of prophylaxis. More importantly, a more detailed mechanistic understanding of RSV transmission dynamics will be crucial to help predict the impact of RSV vaccination programs, as vaccine candidates are currently undergoing clinical trials.

## Materials and Methods

### Data sources

We examined the spatiotemporal pattern of RSV activity in the United States using two sources of weekly epidemiological data: (1) age-specific hospitalizations with any mention of RSV from ten State Inpatient Databases (SIDs) of the Healthcare Cost and Utilization Project (HCUP), from January 1989 to December 2009 for nine states (Arizona, California, Colorado, Iowa, Massachusetts, Maryland, New Jersey, Washington, and Wisconsin) and from 1989–1996 and 2005–2009 for Florida, and (2) laboratory reporting of the number of RSV-positive tests (by any detection method) from the National Respiratory and Enteric Virus Surveillance System (NREVSS), for which 38 states had at least 10 consecutive years of consistent data between July 1989 and May 2010.

#### Hospitalization data

Age-specific data on hospitalizations for RSV were obtained from the State Inpatient Databases (SIDs) of the Healthcare Cost and Utilization Project (HCUP) maintained by the Agency for Healthcare Research and Quality (AHRQ) through an active collaboration with AHRQ. All hospital discharge records from community hospitals in participating states are included in the database. HCUP databases bring together the data collections efforts of State data organizations, hospital associations, private data organizations, and the Federal government to create a national information resource of encounter-level health care data [59]. We extracted all hospitalization records that included the International Classification of Diseases 9^th^ revision, Clinical Modification (ICD-9-CM) code for RSV (079.6, 466.11, 480.1) in any position among up to 15 discharge diagnoses that were consistently available. We extracted data on the age of the patient (in 1-year age categories from 0–4 years old and 5-year age categories from 5–9 years to ≥95 years old), week and year of admission, and hospital state. We also extracted data on hospitalizations for bronchiolitis (ICD-9-CM 466.1) for comparison, but focused our analysis on the RSV-specific hospitalization data; for the purposes of fitting models to the seasonal pattern and age distribution of RSV cases, the specificity of the diagnosis is more important than the sensitivity.

We limited our analysis to the nine states that had data available from January 1, 1989 to December 31, 2009: Arizona, California, Colorado, Iowa, Massachusetts, Maryland, New Jersey, Washington, and Wisconsin. We also included Florida in our analysis because of its unusual seasonal pattern, even though data was not available from 1997–2004.

The RSV-specific ICD-9 code for acute bronchiolitis [466.11] was introduced in September 1996, leading to a large increase in RSV-coded hospitalizations (Fig. 1). To account for this change, we calculated a correction factor for each state equal to the mean number of RSV hospitalizations per week from 1997 to 2009 over the mean number of weekly RSV hospitalizations from 1989 to 1995. We then multiplied the pre-September 1996 hospitalization time series by the state-specific correction factor. Since we had limited data for Florida, we multiplied the pre-September 1996 hospitalization data by the mean under-reporting factor for the other nine states. The adjusted number of RSV-specific hospitalizations was similar to the rate of bronchiolitis hospitalizations in children <5 years old before and after September 1996 (S5 Fig.).

#### Laboratory reporting of RSV

Data on laboratory reporting of RSV tests by state (including the District of Columbia) from July 1989 to May 2010 were obtained from NREVSS. A map and list of current participating laboratories can be found on the NREVSS website (http://www.cdc.gov/surveillance/nrevss/labs/default.html). We included RSV detections by all three diagnostic methods collected in NREVSS: antigen detection, reverse transcription polymerase chain reaction (RT-PCR) and viral culture. While the use of these different diagnostic methods has varied over time, so long as they do not vary seasonally (and in different ways in different states), this variation in testing methods should not bias our analysis. We limited our analysis to states with at least 10 consecutive years of consistent reporting (defined as ≥100 RSV tests, ≥10 RSV-positive samples, and <15 consecutive weeks in which no laboratories reported results to NREVSS annually). The resulting dataset consisted of 38 states; the total number of RSV-positive samples by state ranged from 587 (District of Columbia) to 65,232 (Texas).

We rescaled the laboratory data on the number of RSV-positive tests to account for changes in testing practices over time. First, we calculated a two-year moving average of the weekly number of RSV tests (both positive and negative specimen results) in each state. We then calculated a weekly scaling factor equal to the average number of RSV tests for the state during the entire period of consistent reporting divided by the two-year moving average (S6 Fig.). The rescaled number of RSV-positive tests was then estimated to be the reported number of positive tests times the scaling factor (S6 Fig.). We estimate a “reporting fraction” (*h*) when fitting the dynamic model to the laboratory data (see “Dynamic model description” below); hence, we do not need to know the exact level of reporting so long as it is consistent through time.

In some states, there may have been changes in the geographic distribution of laboratories that report to NREVSS over time. This could have affected the spatiotemporal pattern of epidemics observed within that state, e.g. if there was greater (or less) representation of rural areas over time, which may have slightly different timing of RSV activity than urban centers. However, since we are fitting our model to capture the average timing of epidemics over time (for at least 10 years), the impact on our conclusions regarding the overall spatiotemporal pattern of RSV activity across states should be minimal.

#### Demographic data

The initial population size for each state was obtained from the 1990 US census data [60]. We assumed the birth rate varied between states and over time, according to data on the crude annual birth rate for each state from 1990 to 2009 [61]. Individuals were assumed to age exponentially into the next age class, with the rate of aging equal to 1/(width of the age class). We divided the <1 year age class into 12-month age groups to more accurately capture aging among this important age class (in which >70% of cases occur). The remaining population was divided into 6 classes: 1–4 years old, 5–9 years, 10–19 years, 20–39 years, 40–59-years, and 60+ years old. We assumed deaths occurred from the last age class and adjusted the net rate of immigration/emigration and death from other age groups in order to produce a rate of population growth and age structure similar to that of the US.

#### Climate data

Monthly climatic data were obtained from worldwide climate maps generated by the interpolation of climatic information from ground-based meteorological stations with a monthly temporal resolution and 0.5° latitude by 0.5° longitude spatial resolution (update CRU TS 3.0 0.5°, available from http://badc.nerc.ac.uk/view/badc.nerc.ac.uk__ATOM__dataent_1256223773328276) [62]. The climatic variables used were precipitation, monthly average of daily minimum and maximum temperatures, average temperature, diurnal temperature range, potential evapotranspiration (PET), average number of wet days (days with >1 mm of rain), cloud cover, and vapor pressure. These monthly climatic variables were extracted from the pixels with more than 10,000 inhabitants, within each US state for the period from 1994 to 2004. Weekly time series were derived from the monthly data using linear interpolation. Climatic information was extracted and checked for consistency using scripts written in MATLAB version 7.5.0 (MathWorks, Natick, MA) specifically for this purpose.

As a sensitivity analysis, we also extracted daily data for all weather indicators from the National Oceanic and Atmospheric Administration's North American Regional Reanalysis (http://www.esrl.noaa.gov/psd/data/gridded/data.narr.html). We only considered the 30 by 30 km pixel corresponding to the census-determined population center of each state, and aggregated daily data at the weekly level from January 1, 1989 to December 31, 2010. Despite the different temporal and geographic resolution and data sources, the two datasets were highly correlated (*ρ* = 0.95–0.99 for temperature and PET, *ρ*>0.75 for all other indicators), and the results of our analyses remained qualitatively the same (S2 Table and S8 Table).

### Exploratory analyses of putative environmental drivers

Summary measures describing the seasonal variation in temperature, PET, and vapor pressure, including the mean value, relative amplitude of seasonal fluctuations, and seasonal offset, were derived by fitting harmonic curves to the climatic time series (S5 Fig.). We also calculated mean monthly and weekly values for all climatic variables in each state, and used these to estimate deviations from the average state-specific climatology.

We obtained two complementary measures of RSV epidemic timing, based on the center of gravity (mean epidemic week, where each week is weighted by the number of cases—S1 Text, [36]) and phase decomposition obtained from wavelet analysis [40], [63]. In the wavelet analysis, we used the 0.8–1.2 year periodicity band from the wavelet spectrum to extract weekly phases, and calculated the difference between phase in Florida, where RSV epidemics are earliest, and phases in the other states, averaged throughout the study period.

Following earlier work [36], [64], we estimated the statistical association between empirical seasonal patterns of RSV and climate factors using univariate and stepwise multivariate regression models, with RSV timing as the outcome (center of gravity or phase difference with Florida), and climate variables as predictors. Monthly climate predictors were summarized as annual and seasonal means; since fall (September-November) climate values were most strongly associated with RSV, we do not report regression results for the other seasons here. We also considered demographic (birth rate, population size and density), geographic (latitude, longitude), and sampling (number of RSV tests) factors in multivariate regression models. Finally, time trends in climate and RSV seasonal characteristics were assessed by linear regression using year as a potential predictor.

### Dynamic model description

We developed an age-structured SIRS model to describe the transmission dynamics of RSV (Fig. 2A). The model assumes individuals are born with protective maternal immunity (*M*), which wanes exponentially (with a mean duration of 3–4 months) [65], leaving the infant susceptible to infection (*S_n_*, where *n* is the number of previous infections). Following infection with RSV, individuals develop partial immunity, which reduces the rate of subsequent infection and the duration and relative infectiousness of such infections, consistent with epidemiological studies and previous models of RSV transmission [26], [27], [29]. We assume a progressive build-up of immunity following one, two, and three or more previous infections (*I_n_*) [37], [66]–[68]. Both age and number of previous infections were assumed to influence the risk of developing severe lower respiratory disease (*D*) possibly requiring hospitalization [37], [66], [69]. We parameterized the model based on data from cohort studies conducted in the US and Kenya (Table 2) [37], . Transmission-relevant contact patterns were assumed to be frequency-dependent and were parameterized based on self-reported data on the number and age of conversational partners from one European study [54], [55]; no such study has been conducted among a widely representative cohort in the US.

We initially fit our model to the age-stratified hospitalization data from all nine states with complete data from 1989–2009 in order to estimate the mean transmission rate, relative infectiousness of first and second versus subsequent infections, seasonality parameters, and reporting fraction (i.e. proportion of individuals with severe lower respiratory tract disease who are hospitalized, coded as RSV, and reported in our dataset), which are key unknown parameters. We then fixed the relative infectiousness and fit the model to the hospitalization data from each of the nine states plus Florida individually, using the other estimated parameters from the cumulative data fit as our starting conditions to estimate state-specific transmission rates, seasonality parameters, and reporting fractions. For each fit, we seeded the model with one infectious individual in each age group and used a burn-in period of 40 or 41 years, examining the fit using both even- and odd-year burn-in periods to allow for the biennial pattern of epidemics present in some states, and selected the best-fitting model for each state. We also explored longer burn-in periods and examined the model output to ensure that the equilibrium quasi-steady state had been reached.

Seasonality in the instantaneous rate of transmission of RSV was modeled using sinusoidal seasonal forcing with a period of 1 year (52.18 weeks) as follows: 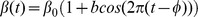, where *β_0_* is the baseline transmission rate, *b* is the amplitude of seasonality, and *φ* is a seasonal offset parameter (a measure of timing of peak transmissibility), and *t* is the time (in years) [31], [32]. We constrained *φ* to be between −0.5 and 0.5, where *φ* = 0 represents January 1 and *φ* = −0.5 and *φ* = 0.5 both represent July 1. These parameters were estimated by fitting our model to the state-specific data.

We used maximum likelihood to determine the best-fitting models. For each set of parameters, the likelihood of the data given the model was calculated by assuming the number of hospitalizations in each age class (*a*) during each week (*w*), *x_a,w_*, was Poisson-distributed with a mean equal to the model-predicted number of severe lower respiratory tract infections due to RSV (*D_a,w_*) times the reporting (or hospitalized) fraction (*h*), 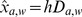, as has been described previously [27], [36]. The log-likelihood (log(*L*)) of the model was given by the equation:
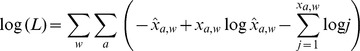



While this observation model may fail to capture the true variability in the distribution of cases, other observation models (e.g. negative binomial) would require estimating an additional parameter, which we do not feel is justified. We used the “fminsearch” command in MATLAB v7.14 (MathWorks, Natick, MA) to minimize the –log(*L*), which employs a direct simplex search method.

Next, we fit the model to the laboratory data on RSV-positive tests from 38 states. The laboratory data did not contain detail on the age of cases; therefore, we could not derive reliable estimates of the baseline transmission rate by fitting our model to these data, since estimates of the baseline transmission rate and reporting fraction are inherently confounded. (The age distribution of cases, pattern of epidemics, and mean incidence rate inform estimates of the baseline transmission rate, while the mean incidence also informs estimates of the reporting fraction.) Instead, we estimated the baseline transmission rate for each state from the relationship we observed between population density and *R*_0_ among the ten states with hospitalization data (S3 Fig.). We fixed the *R*_0_ for the District of Columbia at the maximum observed *R*_0_ (for New Jersey). We then estimated the amplitude of seasonality, seasonal offset parameter, and reporting fraction by fitting our model to the rescaled laboratory data. We examined the sensitivity of our results to the method we used to correct for changes in testing and reporting effort for RSV over time by also fitting our model to the raw number of RSV-positive tests reported, instead applying the estimated scaling factor to the model output (i.e. dividing the model output by the scaling factor for each week). The log-likelihoods of the fitted models were similar (S5 Table), and the results were qualitatively the same (S8 Table).

We examined the correlation between the estimated model parameters for each state and the significant climatic variables from the univariate statistical analyses. We calculated the Pearson's correlation coefficient and associated *p*-value for each state-specific parameter estimate and climatic variable of interest. We also examined the ability of the model to capture the biennial pattern of RSV epidemics present in some states by comparing the strength of the biennial cycle in the observed and predicted RSV time series. The strength of the biennial cycle was calculated as the ratio of the biennial to annual Fourier amplitude [73], [74]. Finally, we examined whether monthly deviations from average climatic conditions could help explain the difference between observed and predicted monthly RSV activity across states.

## Supporting Information

S1 Fig **Fit of transmission dynamic model for RSV to age-specific hospitalization data.** The rescaled weekly hospitalization data (accounting for the change in reporting practices that occurred in September 1996) is shown in blue, while the fitted models are shown in red. Age distributions of RSV hospitalizations among children <5 years of age are also shown for the data and fitted models.
(EPS)

S2 Fig **Plots of weighted univariate regression models of RSV timing against key climatic predictors, where weights are defined as 1/variance in timing estimates.** Timing is based on the center of gravity in RSV activity in weekly laboratory-surveillance reports in 50 states and DC (blue dots), averaged over 21 epidemics 1989–2010 (vertical red bars represent variance). Climate variables are averaged over the fall period for (*A*) vapor pressure (hecta-Pascals), (*B*) minimum temperature (°C), and (*C*) potential evapotranspiration (mm/day). Blue lines represent predicted values and shaded areas represent 95% CI. See Table 1 for parameter estimates.
(TIF)

S3 Fig **Relationship between the basic reproductive number and population density.** The value of *R*_0_ estimated by fitting the transmission dynamic model to the age-specific hospitalization data is plotted against the population density for each state. The dots are color-coded according to the mean center of gravity of RSV activity in each state, as indicated by the color bar.
(EPS)

S4 Fig **Analysis of transmission model residuals.** Deviations from model-predicted patterns (observed minus predicted monthly lab reports for October to April) for 38 states versus (*A*) monthly minimum temperature minus average state-specific minimum temperature for that month, (*B*) monthly vapor pressure minus average vapor pressure, (*C*) monthly precipitation minus average precipitation, and (*D*) monthly potential evapotranspiration minus average potential evapotranspiration at lags of 0 and 1 month between the case time series and the climate time series.
(EPS)

S5 Fig **Seasonal variation in climatic variables.** Average monthly values for the years 1994 to 2004 (solid lines) and sine curves fitted to the monthly averages (dotted lines) are plotted for each of the climate variables for select states.
(EPS)

S6 Fig **Comparison of data on weekly hospitalizations for RSV and acute bronchiolitis.** (*A*) The adjusted number hospitalizations for RSV (ICD-9-CM 079.6, 466.11, 480.1) per week in California (blue) is plotted along with the number of hospitalizations for acute bronchiolitis (ICD-9-CM 466.1) for all age groups (pink) and for children <5 years old (light blue). (*B*) The age distribution of patients <5 years old hospitalized with a diagnosis of RSV (blue) or acute bronchiolitis (light blue). (*C*) The age distribution of patients of all ages hospitalized with a diagnosis of RSV (blue) or acute bronchiolitis (pink).
(EPS)

S7 Fig **Rescaling of laboratory data to account for changes in RSV reporting over time.** (*A*) The number of weekly RSV-positive tests for South Dakota (left) and Florida (right) for the entire period from July 1989 to June 2010 (cyan) and the period with consistent reporting (defined as ≥100 RSV tests/year, ≥10 RSV-positive samples/year, and <15 consecutive weeks/year in which no laboratories reported results to NREVSS) (blue). (*B*) The total number of tests for RSV (positive and negative) by week (blue), along with the 2-year moving average number of tests for each week (red) and mean number of tests per week across the entire period with consistent reporting (green). (*C*) Rescaled number of RSV-positive tests, where the scaling factor is equal to the mean number of tests over the entire period with consistent reporting (green line in *B*) divided by the weekly moving average number of tests (red line in *B*). The rescaled number of weekly RSV hospitalizations for Florida are plotted in grey (on the right axis) for comparison.
(EPS)

S1 Table **Correlation between RSV hospitalizations and laboratory reports (rescaled number of RSV-positive specimens) for states with both types of data.**
(DOCX)

S2 Table **Sensitivity analysis on climate variables: univariate regression of timing of RSV activity against climatic indicators derived from weekly data.** (Same as Table 1 but using weekly climatic data for the population center of each state from the NOAA/NARR reanalysis dataset.) Separate models are built for phase timing (average weekly phase difference with Florida, the earliest RSV state) and center of gravity (weighted average of RSV epidemic week, where each week is weighted by the number of RSV cases). All epidemic measures are based on weekly laboratory-reported RSV time series. Results are provided for climate indicators summarized annually or during the fall period. Boldface indicates significance (*p*<0.05) in the exploratory analysis. As in Table 1, specific humidity (also known as vapor pressure) and temperature and are the strongest univariate predictors of RSV timing. Stepwise multivariate regression consistently indicates that specific humidity is the strongest predictor of RSV timing, consistent with S3 Table.
(DOCX)

S3 Table **Stepwise multivariate regression of RSV timing in 50 US states and District of Columbia.** Two indicators of timing are considered as outcome: phase extracted from the 1-year component of reconstructed wavelet decomposition (average weekly phase difference with Florida; see methods) and center of gravity (see earlier description in supplement). The potential explanatory variables are listed in Table 1. *p*-value for entry<0.20; *p*-value for remaining in model <0.05.
(DOCX)

S4 Table **Correlation among estimated parameters for the transmission dynamic model.**
(DOCX)

S5 Table **Comparison of baseline transmission dynamic model to model with the transmission rate directly proportional to PET.** Log-likelihood of model fit to the laboratory report data from 38 states using sinusoidal seasonal forcing versus modeling the transmission rate as directly proportional to weekly variations in PET.
(DOCX)

S6 Table **Comparison of baseline transmission dynamic model to model including school-term forcing.** Log-likelihood of transmission dynamic model fit to hospitalization data from 10 states using sinusoidal seasonal forcing alone versus including both school-term and sinusoidal seasonal forcing of the transmission rate.
(DOCX)

S7 Table **Time trends in climate variables for the 50 US states plus District of Columbia, 1994-2004 based on the monthly CRU climate dataset.** Only states with significant trends (*p*<0.05) are listed.
(DOCX)

S8 Table **Sensitivity of correlation between seasonality parameters and climatic variables to method of rescaling laboratory data and resolution of climate data.** Correlation coefficients between estimated seasonality parameters of model fit to laboratory data with scaling factor applied to model output and sine curves fit to the monthly (CRU) and weekly (NOAA) climate data.
(DOCX)

S1 Text **Supplementary methods.**
(PDF)
