# Supplementary figures and images for: Environmental Drivers of the Spatiotemporal Dynamics of Respiratory Syncytial Virus in the United States
Source: PLoS Pathog. 2015 Jan 8;11(1):e1004591. doi: 10.1371/journal.ppat.1004591 (PMC4287610; doi:10.1371/journal.ppat.1004591)

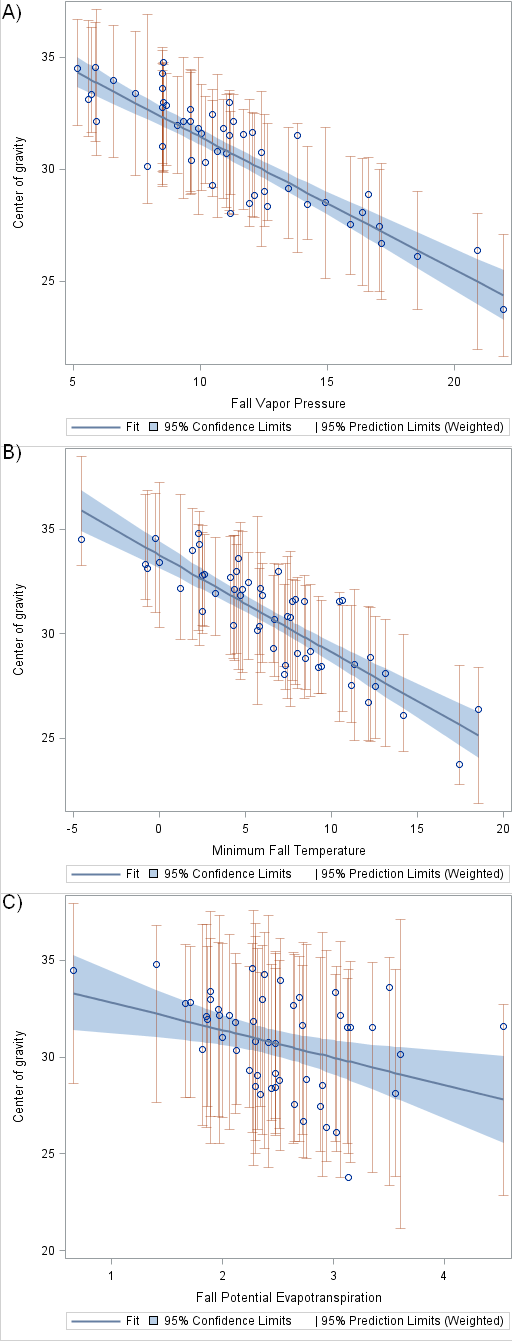

Supplement: S2 Fig — Plots of weighted univariate regression models of RSV timing against key climatic predictors, where weights are defined as 1/variance in timing estimates. Timing is based on the center of gravity in RSV activity in weekly laboratory-surveillance reports in 50 states and DC (blue dots), averaged over 21 epidemics 1989–2010 (vertical red bars represent variance). Climate variables are averaged over the fall period for (A) vapor pressure (hecta-Pascals), (B) minimum temperature (°C), and (C) potential evapotranspiration (mm/day). Blue lines represent predicted values and shaded areas represent 95% CI. See Table 1 for parameter estimates. (TIF) [file ppat.1004591.s002.tif]
