# Supplementary material for: Environmental Drivers of the Spatiotemporal Dynamics of Respiratory Syncytial Virus in the United States
Source: PLoS Pathog. 2015 Jan 8;11(1):e1004591. doi: 10.1371/journal.ppat.1004591 (PMC4287610; doi:10.1371/journal.ppat.1004591)
Supplement: S1 Table — Correlation between RSV hospitalizations and laboratory reports (rescaled number of RSV-positive specimens) for states with both types of data. (DOCX) [file ppat.1004591.s008.docx]

**Table S1. Correlation between RSV hospitalizations and laboratory reports (rescaled number of RSV-positive specimens) for states with both types of data.**

| **State** | **Correlation coefficient** |
| --- | --- |
| Arizona | 0.861 |
| California | 0.922 |
| Colorado | 0.807 |
| Florida | 0.886 |
| Illinois | 0.751 |
| Massachusetts | 0.722 |
| New Jersey | 0.719 |
| Washington | 0.865 |
| Wisconsin | 0.844 |
