# Supplementary material for: Environmental Drivers of the Spatiotemporal Dynamics of Respiratory Syncytial Virus in the United States
Source: PLoS Pathog. 2015 Jan 8;11(1):e1004591. doi: 10.1371/journal.ppat.1004591 (PMC4287610; doi:10.1371/journal.ppat.1004591)
Supplement: S2 Table — Sensitivity analysis on climate variables: univariate regression of timing of RSV activity against climatic indicators derived from weekly data. (Same as Table 1 but using weekly climatic data for the population center of each state from the NOAA/NARR reanalysis dataset.) Separate models are built for phase timing (average weekly phase difference with Florida, the earliest RSV state) and center of gravity (weighted average of RSV epidemic week, where each week is weighted by the number of RSV cases). All epidemic measures are based on weekly laboratory-reported RSV time series. Results are provided for climate indicators summarized annually or during the fall period. Boldface indicates significance (p<0.05) in the exploratory analysis. As in Table 1, specific humidity (also known as vapor pressure) and temperature and are the strongest univariate predictors of RSV timing. Stepwise multivariate regression consistently indicates that specific humidity is the strongest predictor of RSV timing, consistent with S3 Table. (DOCX) [file ppat.1004591.s009.docx]

**Table S2. Sensitivity analysis: univariate regression of timing of RSV activity in 50 US states and District of Columbia, 1989-2010, against weekly climatic indicators.** (Same as Table 1 but using weekly climatic data for the population center of each state from the NOAA/NARR reanalysis dataset.) Separate models are built for phase timing (average weekly phase difference with Florida, the earliest RSV state) and center of gravity (weighted average of RSV epidemic week, where each week is weighted by the number of RSV cases). All epidemic measures are based on weekly laboratory-reported RSV time series. Results are provided for climate indicators summarized annually or during the fall period. Boldface indicates significance (*p*<0.05) in the exploratory analysis. As in Table 1, specific humidity (also known as vapor pressure) and temperature and are the strongest univariate predictors of RSV timing. Stepwise multivariate regression consistently indicates that specific humidity is the strongest predictor of RSV timing, consistent with Table S3.

|  | Outcome= RSV phase timing | | | | Outcome= RSV center of gravity | | | |
| --- | --- | --- | --- | --- | --- | --- | --- | --- |
| Explanatory variable | Parameter estimate (SE) | R^2^ | Parameter estimate (SE) | R^2^ | Parameter estimate (SE) | R^2^ | Parameter estimate (SE) | R^2^ |
| *Climate variables:* | Annual average | | Fall average^a^ | | Annual average | | Fall average | |
| **Specific Humidity ^b^** | **-91.5*** (10.1)** | **62%** | **-91.8*** (9.3)** | **66%** | **-829.7*** (79.4)** | **68%** | **-824. 7*** (73.7)** | **71%** |
| **Minimum Temperature^c^** | **-0.039*** (0.005)** | **52%** | **-0.039*** (0.005)** | **52%** | **-0.361*** (0.042)** | **59%** | **-0.335*** (0.042)** | **60%** |
| Precipitation | -0.0099 (0.0055) | 4% | -0.0047 (0.005) | 0% | **-0.113* (0.047)** | **9%** | -0. 059*** (0.045) | 1% |
| Potential evapotranspiration | -0.03 (0.02) | 2% | **-0.057* (0.024)** | **8%** | -0.312 (0.195) | 3% | **-0.540* (0.208)** | **10%** |
| Wet days | -0.066 (0.039) | 0% | -0.025 (0.066) | 0% | -0. 642 (0.592) | 0% | -0.159 (0. 570) | 0% |
| Cloud cover | -0.006 (0.004) | 2% | -0.0038 (0.0038) | 0% | -0.048 (0.036) | 0% | -0.027 (0.033) | 0% |
| Diurnal temperature range | 0.012 (0.021) | 0% | 0.011 (0.019) | 0% | 0.093 (0.179) | 0% | 0.060 (0.166) | 0% |

* P<0.05; ** P<0.01; *** P<0.0001

^a^ Average value for September, October, and November (weeks 36-48).

^b^ In Table 1, we use vapor pressure from the NCAR dataset, while here we use specific humidity from the NOAA/NARR dataset. Vapor pressure and specific humidity represent the same climatic parameter using different units, explaining the differences in estimated regression coefficients reported in Tables 1 and S2. Nevertheless, across all analyses, vapor pressure/specific humidity were the strongest predictors of state-specific RSV timing.

^c^ Minimum, maximum and average weekly temperatures were considered. While all temperature variables were associated with RSV timing and yielded similar parameter values and significance level, the strongest association was always with minimum temperature. We report results for minimum temperature here.
