# Supplementary material for: Environmental Drivers of the Spatiotemporal Dynamics of Respiratory Syncytial Virus in the United States
Source: PLoS Pathog. 2015 Jan 8;11(1):e1004591. doi: 10.1371/journal.ppat.1004591 (PMC4287610; doi:10.1371/journal.ppat.1004591)
Supplement: S3 Table — Stepwise multivariate regression of RSV timing in 50 US states and District of Columbia. Two indicators of timing are considered as outcome: phase extracted from the 1-year component of reconstructed wavelet decomposition (average weekly phase difference with Florida; see methods) and center of gravity (see earlier description in supplement). The potential explanatory variables are listed in Table 1. p-value for entry<0.20; p-value for remaining in model <0.05. (DOCX) [file ppat.1004591.s010.docx]

**Table S3. Stepwise multivariate regression of RSV timing in 50 US states and District of Columbia.** Two indicators of timing are considered as outcome: phase extracted from the 1-year component of reconstructed wavelet decomposition (average weekly phase difference with Florida; see methods) and center of gravity (see earlier description in supplement). The potential explanatory variables are listed in Table 1. *p*-value for entry<0.20; *p*-value for remaining in model <0.05.

|  | **Phase timing indicator** | | **Center of gravity indicator** | |
| --- | --- | --- | --- | --- |
| Selected variable(s) | Parameter estimate (SE) | Partial R^2^ | Parameter estimate (SE) | Partial R^2^ |
| Fall vapor pressure | -0.064*** (0.006) | 72% | -0.575*** (0.045) | 77% |

* *p*<0.05; ** *p*<0.01; *** *p*<0.0001
