# Supplementary material for: Environmental Drivers of the Spatiotemporal Dynamics of Respiratory Syncytial Virus in the United States
Source: PLoS Pathog. 2015 Jan 8;11(1):e1004591. doi: 10.1371/journal.ppat.1004591 (PMC4287610; doi:10.1371/journal.ppat.1004591)
Supplement: S4 Table — Correlation among estimated parameters for the transmission dynamic model. (DOCX) [file ppat.1004591.s011.docx]

**Table S4. Correlation among estimated parameters for the transmission dynamic model.**

|  | **Hospitalization data** | | | | **Laboratory data** | | |
| --- | --- | --- | --- | --- | --- | --- | --- |
| Parameter | *R*_0_ | *b* | *φ* | *h* | *b* | *φ* | *h* |
| *R*_0_ | 1 | -0.55 | -0.69* | -0.37 | NA^†^ | NA^†^ | NA^†^ |
| *b* | -0.55 | 1 | 0.88** | 0.21 | 1 | 0.74*** | 0.43** |
| *φ* | -0.69* | 0.88** | 1 | 0.33 | 0.74*** | 1 | 0.37* |
| *h* | -0.37 | 0.21 | 0.33 | 1 | 0.43** | 0.37* | 1 |

* *p*<0.05; ** *p*<0.01; *** *p*<0.0001

^†^*R*_0_ was not estimated from laboratory data due to lack of age detail.
