# Supplementary material for: Environmental Drivers of the Spatiotemporal Dynamics of Respiratory Syncytial Virus in the United States
Source: PLoS Pathog. 2015 Jan 8;11(1):e1004591. doi: 10.1371/journal.ppat.1004591 (PMC4287610; doi:10.1371/journal.ppat.1004591)
Supplement: S5 Table — Comparison of baseline transmission dynamic model to model with the transmission rate directly proportional to PET. Log-likelihood of model fit to the laboratory report data from 38 states using sinusoidal seasonal forcing versus modeling the transmission rate as directly proportional to weekly variations in PET. (DOCX) [file ppat.1004591.s012.docx]

**Table S5. Comparison of baseline transmission dynamic model to model with the transmission rate directly proportional to PET.** Log-likelihood of model fit to the laboratory report data from 38 states using sinusoidal seasonal forcing versus modeling the transmission rate as directly proportional to weekly variations in PET.

| **State (Abbreviation)** | **Log-likelihood of model** | | |
| --- | --- | --- | --- |
|  | Sinusoidal forcing (fit rescaled data) | Sinusoidal forcing (scaling factor applied to model) | Proportional to PET (scaling factor applied to model) |
| Alabama (AL) | -2,910 | -2,378 | -5,921 |
| Arizona (AZ) | -4,832 | -5,978 | -9,895 |
| Arkansas (AR) | -1,777 | -1,636 | -3,763 |
| California (CA) | -8,421 | -7,204 | -24,603 |
| Colorado (CO) | -3,787 | -3,638 | -8,744 |
| Delaware (DE) | -1,791 | -1,921 | -4,605 |
| District of Columbia (DC) | -831 | -786 | -989 |
| Florida (FL) | -5,570 | -3,390 | -13,180 |
| Georgia (GA) | -7,676 | -4,653 | -21,098 |
| Hawaii (HI) | -3,320 | -3,252 | -4,840 |
| Illinois (IL) | -5,115 | -5,356 | -14,336 |
| Indiana (IN) | -4,370 | -7,467 | -11,513 |
| Kentucky (KY) | -5,063 | -5,109 | -8,576 |
| Louisiana (LA) | -5,408 | -6,261 | -19,389 |
| Massachusetts (MA) | -1,463 | -1,084 | -2,218 |
| Michigan (MI) | -1,121 | -1,235 | -2,271 |
| Minnesota (MN) | -2,146 | -2,530 | -8,819 |
| Mississippi (MS) | -1,526 | -1,537 | -2,532 |
| Missouri (MO) | -8,289 | -8,129 | -24,685 |
| Montana (MT) | -1,564 | -1,831 | -3,835 |
| Nebraska (NE) | -2,645 | -3,266 | -7,256 |
| Nevada (NV) | -3,189 | -3,371 | -4,843 |
| New Hampshire (NH) | -1,351 | -1,505 | -1,993 |
| New Jersey (NJ) | -3,498 | -2,744 | -9,516 |
| New York (NY) | -8,071 | -6,838 | -24,226 |
| North Carolina (NC) | -2,926 | -2,406 | -5,514 |
| North Dakota (ND) | -2,020 | -1,999 | -2,945 |
| Ohio (OH) | -5,563 | -4,179 | -13,966 |
| Oklahoma (OK) | -3,535 | -3,419 | -6,681 |
| South Carolina (SC) | -2,291 | -2,500 | -5,368 |
| South Dakota (SD) | -3,303 | -4,059 | -11,148 |
| Tennessee (TN) | -5,814 | -5,289 | -13,402 |
| Texas (TX) | -13,886 | -8,129 | -45,966 |
| Virginia (VA) | -5,169 | -3,893 | -9,147 |
| Washington (WA) | -4,405 | -6,660 | -11,120 |
| West Virginia (WV) | -2,217 | -2,871 | -5,737 |
