# Supplementary material for: Environmental Drivers of the Spatiotemporal Dynamics of Respiratory Syncytial Virus in the United States
Source: PLoS Pathog. 2015 Jan 8;11(1):e1004591. doi: 10.1371/journal.ppat.1004591 (PMC4287610; doi:10.1371/journal.ppat.1004591)
Supplement: S6 Table — Comparison of baseline transmission dynamic model to model including school-term forcing. Log-likelihood of transmission dynamic model fit to hospitalization data from 10 states using sinusoidal seasonal forcing alone versus including both school-term and sinusoidal seasonal forcing of the transmission rate. (DOCX) [file ppat.1004591.s013.docx]

**Table S6. Comparison of baseline transmission dynamic model to model including school-term forcing.** Log-likelihood of transmission dynamic model fit to hospitalization data from 10 states using sinusoidal seasonal forcing alone versus including both school-term and sinusoidal seasonal forcing of the transmission rate.

| **State (Abbreviation)** | **Log-likelihood of model** | |
| --- | --- | --- |
|  | Sinusoidal forcing only | School-term & sinusoidal forcing |
| Arizona (AZ) | -20,098 | -41,774 |
| California (CA) | -47,366 | -129,010 |
| Colorado (CO) | -17,710 | -26,118 |
| Iowa (IA) | -11,707 | -16,625 |
| Massachusetts (MA) | -14,388 | -19,519 |
| Maryland (MD) | -18,307 | -29,140 |
| New Jersey (NJ) | -15,771 | -16,470 |
| Washington (WA) | -14,135 | -21,841 |
| Wisconsin (WI) | -13,120 | -18,369 |
| Florida (FL) | -16,372 | -18,196 |
