# Supplementary material for: Environmental Drivers of the Spatiotemporal Dynamics of Respiratory Syncytial Virus in the United States
Source: PLoS Pathog. 2015 Jan 8;11(1):e1004591. doi: 10.1371/journal.ppat.1004591 (PMC4287610; doi:10.1371/journal.ppat.1004591)
Supplement: S7 Table — Time trends in climate variables for the 50 US states plus District of Columbia, 1994-2004 based on the monthly CRU climate dataset. Only states with significant trends (p<0.05) are listed. (DOCX) [file ppat.1004591.s014.docx]

**Table S7. Time trends in climate variables based on the monthly CRU dataset for the 50 US states plus District of Columbia, 1994-2004.** Only states with significant trends (*p*<0.05) are listed.

| Climate variable | States experiencing **increasing** climate trend | | States experiencing **decreasing** climate trend | |
| --- | --- | --- | --- | --- |
|  | Fall climate (Oct-Dec) | Year-round climate | Fall climate (Oct-Dec) | Year-round climate |
| Vapor pressure | AK | None | None | HI, ID, MT, OR, WA |
| Minimum temperature | AK | AK, KY, NV, VA, WV | None | None |
| Average temperature | AK | AK | None | None |
| Maximum temperature | None | SD | None | NM |
| Precipitation | None | None | ID, MT, SC | WA |
| Wet days | None | None | ID | None |
| Potential evapo-transpiration | OR, WA | SD | None | IN, NM, NY, PA |
| Cloud cover | CO, IN, NJ | None | GA, SC | Most states |
| Diurnal temperature range | FL, GA, SC, NC | 2 states | AK, NM | 12 states |
