# Supplementary material for: Environmental Drivers of the Spatiotemporal Dynamics of Respiratory Syncytial Virus in the United States
Source: PLoS Pathog. 2015 Jan 8;11(1):e1004591. doi: 10.1371/journal.ppat.1004591 (PMC4287610; doi:10.1371/journal.ppat.1004591)
Supplement: S8 Table — Sensitivity of correlation between seasonality parameters and climatic variables to method of rescaling laboratory data and resolution of climate data. Correlation coefficients between estimated seasonality parameters of model fit to laboratory data with scaling factor applied to model output and sine curves fit to the monthly (CRU) and weekly (NOAA) climate data. (DOCX) [file ppat.1004591.s015.docx]

**Table S8. Sensitivity of correlation between seasonality parameters and climatic variables to method of rescaling laboratory data and resolution of climate data.** Correlation coefficients between estimated seasonality parameters of model fit to laboratory data with scaling factor applied to model output and sine curves fit to the monthly (CRU) and weekly (NOAA/NARR) climate data.

|  | **Monthly climate data** | | **Weekly climate data** | |
| --- | --- | --- | --- | --- |
| **Climatic variable** | *Amplitude of seasonality (b)* | *Seasonal offset (φ)* | *Amplitude of seasonality (b)* | *Seasonal offset (φ)* |
| Vapor pressure/Specific humidity | | | | |
| *Mean* | -0.670*** | -0.837*** | -0.721*** | -0.819*** |
| *Amplitude* | -0.216 | -0.470* | -0.304 | -0.379 |
| *Offset* | -0.324 | -0.040 | 0.574** | 0.353 |
| Minimum temperature | | | | |
| *Mean* | -0.634*** | -0.735*** | -0.558** | -0.630*** |
| *Amplitude* | 0.412 | 0.349 | 0.474* | 0.399 |
| *Offset* | -0.360 | -0.083 | -0.398 | -0.223 |
| Precipitation | | | | |
| *Mean* | -0.670*** | -0.707*** | -0.409 | -0.433* |
| *Amplitude* | -0.055 | 0.070 | -0.066 | 0.140 |
| *Offset* | 0.166 | 0.119 | 0.098 | 0.267 |
| Potential evapotranspiration | | | | |
| *Mean* | -0.049 | -0.133 | 0.149 | -0.038 |
| *Amplitude* | 0.627*** | 0.668*** | 0.649*** | 0.626*** |
| *Offset* | 0.510* | 0.725*** | 0.630*** | 0.771*** |
| Wet days | | | | |
| *Mean* | -0.517** | -0.256 | -0.360 | -0.377 |
| *Amplitude* | -0.136 | 0.085 | -0.233 | -0.408 |
| *Offset* | -0.130 | -0.208 | -0.145 | 0.132 |
| Cloud cover | | | | |
| *Mean* | -0.157 | -0.006 | -0.525** | -0.386 |
| *Amplitude* | 0.411 | 0.538** | 0.454* | 0.562** |
| *Offset* | -0.547** | -0.686*** | -0.515** | -0.672*** |
| Diurnal temperature range | | | | |
| *Mean* | 0.483* | 0.354 | 0.369 | 0.175 |
| *Amplitude* | 0.434* | 0.525** | 0.522** | 0.513** |
| *Offset* | 0.464* | 0.652*** | 0.096 | 0.449* |

**p*<0.01, ***p*<0.001, ****p*<0.0001
