# Supplementary material for: Environmental Drivers of the Spatiotemporal Dynamics of Respiratory Syncytial Virus in the United States
Source: PLoS Pathog. 2015 Jan 8;11(1):e1004591. doi: 10.1371/journal.ppat.1004591 (PMC4287610; doi:10.1371/journal.ppat.1004591)
Supplement: S1 Text — Supplementary methods. (PDF) [file ppat.1004591.s016.pdf]

Text S1

Environmental Drivers of the Spatiotemporal Dynamics of Respiratory Syncytial Virus in the United States

Virginia E. Pitzer[1,2], Cécile Viboud[2], Wladimir J. Alonso[2], Tanya Wilcox[2], C. Jessica Metcalf[3,4], Claudia A. Steiner[5], Amber K. Haynes[6], Bryan T. Grenfell[2,4]

[1]*Department of Epidemiology of Microbial Diseases, Yale School of Public Health, Yale University, New Haven, CT USA*

[2]*Fogarty International Center, National Institutes of Health, Bethesda, MD USA*

[3]*Department of Zoology, University of Oxford, Oxford, UK*

[4] *Department of Ecology and Evolutionary Biology, Princeton University, Princeton, NJ USA*

[5]*Healthcare Cost and Utilization Project, Center for Delivery, Organization and Markets, Agency for Healthcare Research and Quality, US Department of Health and Human Services, Rockville, MD, USA*

[6]*Epidemiology Branch, Division of Viral Diseases, National Center for Immunization and Respiratory Diseases, Centers for Disease Control and Prevention, Atlanta, GA, USA*

Contents

1. Calculation of the center of gravity of RSV activity
2. Model equations
3. Calculation of the basic reproductive number (R_0)
4. Changes in RSV center of gravity over time
5. Model including school-term forcing
6. Modeling RSV transmission as directly proportional to PET
7. Supplementary references

1. Calculation of the center of gravity of RSV activity

The timing of RSV epidemics was measured by calculating the center of gravity (G), which was defined by the mean week of RSV activity for each season and epidemic year, as follows:

$$G_{s,y} = \sum_{w\in[1,52]} w * cases_{s,y,w} \Big/ \sum_{w\in[1,52]} cases_{s,y,w},$$

where $G_{s,y}$ is the mean timing of RSV activity for state s and epidemic year y, w is an index for the week of year, where week 1 indicates the first full week of July and week 52 is the last full week of June, $cases_{s,y,w}$ is the number of RSV-positive laboratory tests reported in state s during epidemic year y and week w. Hence, G is the mean week of the epidemic, where each week is weighted by the number of RSV cases.

2. Model equations

The set of differential equations describing our mathematical model for the transmission dynamics of RSV are as follows:

$$\frac{dM_a}{dt} = B_a(t) - (\omega + \mu_a)M_a$$

$$\frac{dS_{0,a}}{dt} = \omega M_a - \lambda_a(t)S_{0,a} - \mu_a S_{0,a}$$

$$\frac{dI_{1,a}}{dt} = \lambda_a(t)S_{0,a} - (\gamma_1 + \mu_a)I_{1,a}$$

$$\frac{dS_{1,a}}{dt} = \gamma_1 I_{1,a} - \sigma_1 \lambda_a(t)S_{1,a} - \mu_a S_{1,a}$$

$$\frac{dI_{2,a}}{dt} = \sigma_1 \lambda_a(t)S_{1,a} - (\gamma_2 + \mu_a)I_{2,a}$$

$$\frac{dS_{2,a}}{dt} = \gamma_2 I_{2,a} - \sigma_2 \lambda_a(t)S_{2,a} - \mu_a S_{2,a}$$

$$\frac{dI_{3,a}}{dt} = \sigma_2 \lambda_a(t)S_{2,a} - (\gamma_3 + \mu_a)I_{3,a}$$

$$\frac{dS_{3,a}}{dt} = \gamma_3 I_{3,a} + \gamma_4 I_{4,a} - \sigma_3 \lambda_a(t)S_{3,a} - \mu_a S_{3,a}$$

$$\frac{dI_{4,a}}{dt} = \sigma_3 \lambda_a(t)S_{3,a} - (\gamma_4 + \mu_a)I_{4,a}$$

where

$$\lambda_a(t) = \sum_i \beta_{a,i}(t)\left(I_{1,i} + \rho_1 I_{2,i} + \rho_2 I_{3,i} + \rho_2 I_{4,i}\right).$$

The model parameters are defined in Table 2 of the main text.

We assume that only first and second infections can result in severe lower respiratory disease (D), and cases reported in the hospitalization and laboratory data ($H_{a,w}$) are proportional to $D_{a,w}$. The probability ($d_{n,a}$) is dependent upon both age and number of previous infections. Note that D and H represent disease states and not infection states, and thus are not included in the differential equations.

3. Calculation of the basic reproductive number (R_0)

We calculated the basic reproductive number, R_0, for our model using the next generation matrix method of van den Driessche and Watmough[1]. The basic reproductive number (R_0) is equal to the maximum eigenvalue of the next generation matrix, FV^{-1}, where

$$F = \begin{pmatrix} \beta_0 & \rho_1\beta_0 & \rho_2\beta_0 & \rho_2\beta_0 \\ 0 & 0 & 0 & 0 \\ 0 & 0 & 0 & 0 \\ 0 & 0 & 0 & 0 \end{pmatrix}$$

and

$$V = \begin{pmatrix} \gamma_1 & 0 & 0 & 0 \\ 0 & \gamma_2 & 0 & 0 \\ 0 & 0 & \gamma_3 & 0 \\ 0 & 0 & 0 & \gamma_3 \end{pmatrix}$$

Therefore,

$$FV^{-1} = \begin{pmatrix} \mathbf{R}_0 & * & * & * \\ 0 & 0 & 0 & 0 \\ 0 & 0 & 0 & 0 \\ 0 & 0 & 0 & 0 \end{pmatrix}$$

where $\mathbf{R_0} = \boldsymbol{\beta_0}/\gamma_1$. Note that $\boldsymbol{\beta_0}$ is a matrix denoting the product of the probability of transmission given contact, q, and the age-specific mixing matrix with entries $C_{i,j}$ equal to the number of contacts (i.e. self-reported conversational partners) between individuals of ages i and j in a typical week, scaled by the proportion of the

population within each age class. Thus, R_0 is the maximum eigenvalue of the age-specific $\mathbf{R_0}$ matrix.

4. Changes in RSV center of gravity over time

Five states experienced a shift in center of gravity of at least 2 weeks, all towards earlier epidemics—Arkansas, Hawaii, Montana, Nevada, and Washington. Of these, only Nevada experienced significant warming (Table S3). Hawaii, Montana, and Washington had a decreasing trend in vapor pressure (Table S3), which should lead to later epidemics, if anything, according to the relationship we observed between vapor pressure and the timing of RSV activity/seasonal forcing.

5. Model including school-term forcing

We examined whether a combination of school-term forcing and sinusoidal variation in the transmission rate could better explain the observed spatiotemporal pattern of RSV activity across states. Our hypothesis was that school-term forcing may be more important in states with a lower amplitude of sinusoidal seasonal forcing, such as Florida, and therefore lead to an earlier epidemic in the fall when school resumes in such states. To incorporate school-term forcing, we assumed that the transmission rate among school-aged children (5-20 years of age) were reduced by 60% and that the transmission rate among pre-school-aged children were reduced by 30% during the summer (weeks 23-34) and winter (weeks 1 and 52)

holidays, in line with recent estimates from the UK [2]. For preliminary analyses, we ignored the slight variation in school terms across the different US states [3]. We refit the model to the hospitalization data from 10 states allowing for both school-term forcing and sinusoidal variation in the transmission rate, and compared the log-likelihood of the model fits to those that did not include school-term forcing.

The model including both school-term forcing and sinusoidal variation in the transmission rate of RSV provided a poorer fit to the hospitalization data from all 10 states (Table S4). In some cases, the fit was considerably worse. For example, the fitted model for California including both school-term and sinusoidal forcing exhibited a stronger biennial pattern and older age distribution of cases than was observed. Furthermore, the geographic variability in the estimated seasonal offset parameters was essentially unchanged by including school-term forcing. Therefore, we did not pursue this hypothesis further.

6. Modeling RSV transmission as directly proportional to PET

We also explored whether we could explain the spatiotemporal pattern of RSV epidemics across different states by directly parameterizing the transmission rate using the weekly climate data for potential evapotranspiration (PET). We assumed that the transmission parameter, $\beta_s(t)$, was directly proportional to weekly data on variations in PET for each state s:

$$\beta_s(t) = \beta_{0,s}\left(1 + b_{PET,s}\left(PET_s(t) - P\bar{E}T_s\right)\right)$$

where $\beta_{0,s}$ is the baseline transmission rate for state s (as described in the main text), $PET_s(t)$ is the potential evapotranspiration in state s during week t calculated from the weekly climate data, $P\bar{E}T_s$ is the mean potential evapotranspiration for state s over all weeks from January 1, 1989 to December 31, 2010, and $b_{PET,s}$ is the proportionality constant, which we estimated for each state by fitting the model to the laboratory report data for all 38 states. We fit the model to the laboratory report data by applying the estimated scaling factor to the model output (rather than rescaling the data itself), as described in the main text.

If RSV transmission was directly related to PET, we would expect our estimates of b_{PET} to similar or the same across all states, or possibly correlated with the mean value of PET for each state. Furthermore, we would expect our model to provide a similar or even slightly better fit to the data compared to our original assumption that $\beta(t)$ varies sinusoidally in a manner that is the same every year. However, we found that the estimates of b_{PET} varied somewhat substantially from state to state (from -0.0056 for Virginia to -0.0523 for Hawaii, with a mean value of -0.0149). This variation was positively correlated with mean PET, but only weakly (ρ=0.485, p=0.002). Furthermore, the fit of the model to the data was worse for all states, and in some cases was substantially worse (e.g. for Florida, Georgia, and Texas), as indicated by the log-likelihood (Table S6). Therefore, it is likely that the relationship between climate and RSV transmission is more complex than can be captured directly by PET (i.e. non-linear or threshold effects), or PET is a proxy for something else that affects RSV transmission.

7. Supplementary References

1. Van den Driessche P, Watmough J (2002) Reproduction numbers and sub-threshold endemic equilibria for compartmental models of disease transmission. Math Biosci 180: 29–48.

2. Eames KTD, Tilston NL, Brooks-Pollock E, Edmunds WJ (2012) Measured dynamic social contact patterns explain the spread of H1N1v influenza. PLoS Comput Biol 8: e1002425. doi:10.1371/journal.pcbi.1002425.

3. Chao DL, Halloran ME, Longini IM (2010) School opening dates predict pandemic influenza A(H1N1) outbreaks in the United States. J Infect Dis 202: 877–880. doi:10.1086/655810.
